# Saccharated Calomel

**Published:** 1873-08

**Authors:** Robert Battey

**Affiliations:** Rome, Ga.


### SACCHARATED CALOMEL.

BY ROBERT BATTEY, M.D., ROME, GA.

RECIPE.—Take of calomel six grains, of refined cane sugar twelve grains—mix in a suitable mortar, and grind *long and diligently,* until the two are thoroughly combined in impalpable powder. Divide into twelve powders, and direct one for the dose, to be placed upon the tongue of the patient.

CASES.—Mrs. N., aged twenty-one, married, has an infant eight months old, was seen in December, 2870, with epileptiform convulsions, much portal congestion, bowels constipated, stomach very irritable and intolerant of remedies. The above powder was directed every six honrs, and four of them, containing in all two grains of the calomel, evacuated the bowels copiously, disgorged bile freely, and apparently terminated the convulsions.

Mrs. L., aged thirty-six, married, has several children, endometritis, nervous system very much racked, suffers habitually from constipation, and uses, at short intervals, the above powder as a purgative. She reports it as being "too strong for her," and divides the dose. Half the powder, representing one-fourth grain calomel, moves the bowels twice, and even three times. The evacuations, she says, are "bilious."

Miss M., aged twenty-six, has obstinate constipation, was seen once only in January, 1871. Directed a mixture of bromide potass, also the saccharated calomel once daily until the bowels should be thoroughly evacuated. Twelve days elapsed without any movement of the bowels, and slight ptyalism ensued.

Mrs. H., aged thirty, married, endometritis with constipation. She uses the saccharated calomel, when needed, as a purgative. One dose always gives two and sometimes three "bilious evacuations."

Capt. S., aged thirty-five, complains of constipation occasionally, with dull, heavy headache—thinks he is "bilious." He has taken the sacchareted calomel repeatedly, and with somewhat variable results. Sometimes it purges very nicely with but a single dose; at another time, he took it four successive nights with very little effect. On the 16th October he took one at night, and another the next morning. The two purged the bowels five times and copiously. He says that he "passed a great deal of bile, and feels somewhat weakened down by it."

Mrs. B., aged forty, married, is in good health, excepting constipation. She uses the powders as occasion requires; never has to take a second dose. It operates from one to three times, very pleasantly. "Evacuations are always bilious."

Mr. H., aged thirty-five, general health rather feeble, but does not resort to medicine, except an occasional purgative. On the 20th November, took one powder at bed-time; had four bilious evacuations next day. Complains that he was weakened by the purging.

Mrs. B., aged twenty-two, young infant, constipation, took a powder at bed-time; bowels purged four times next morning.

Mr. W., aged thirty-three, remittent fever, bowels constipated, took a dose at night, had four evacuations, "very bilious."

Miss B., aged sixteen, catarrhal fever, constipation, took a dose at noon; bowels moved three times during the night.

Mr. H., aged forty-one, catarrh, bowels regular, took a powder; moved the bowels six times through the night and the following day. Says the remedy was too strong for him." "Dark, bilious evacuations."

Master R., aged five years, croup, took a powder at night. Did not move the bowels—required oil the next day.

Master W., aged three, croup, took a powder at night, which operated once with nausea, (an unusual occurrence,) "a large bilious evacuation."

Mrs. B., aged twenty-two, constipation, took a powder at noon—no action; another at bed-time—bowels moved twice during the night.

Master R., aged ten, pleuritis, constipation, took a powder at night, another in the morning, and again at noon. No action—required oil.

Mrs. H., aged sixty-three, ununited fracture of cervix femoris, sedentary life, dyspepsia, torpid bowels, uses purgatives regularly. She finds much comfort from the saccharated calomel; it moves her bowels mildly, and "helps her stomach and liver."

Mrs. S., age forty-six, married, phthisis pulmonalis, is distressingly nauseated by mercurials, even by small doses of blue mass, took a powder at bed-time. Moved the bowels twice, with moderate nausea, but no vomiting. She did not suspect the presence of a mercurial in the powder.

Mr. S., aged thirty, epididymitis, constipation, took a powder in the morning, and had, during the afternoon, three bilious evacuations.

Maj. J., aged thirty-five, subacute bronchitis, took a powder in the morning, and had one copious evacuation at night. Two days subsequently he took another, which failed to act, but an additional dose the following morning moved twice and freely.

Mr. B., aged forty-three, acute catarrh, supervening upon pulmonary tuberculosis, took a powder in the morning, and was moved three times during the night. A week after this, he repeated the dose at night, which gave three full, bilious evacuations next day, and one of same character the following morning.

Miss H., aged twenty-one, sick-headache, constant nausea and vomiting, stomach will not tolerate a sip of water, bowels not moved for five days. The little powder was put upon the tongue in the morning, and repeated again at noon—all food and drink forbidden. During the afternoon and night the bowels were copiously evacuated, and the patient greatly relieved and comforted.

Miss R., aged two, acute catarrh, took a powder at night, another in the morning, and still required oil to move the bowels.

Mrs. G., aged thirty, married, has children, excellent health, except habitual constipation. Ordered a powder at bed-time, to be repeated in the morning, if necessary. She reports that she did not require the second dose, as "the first one moved her most abundantly."

Remarks.—The cases above cited, though somewhat numerous, comprise but a small part of the number who have used the remedy, but they have been selected and grouped together as offering a fair exhibition of its usual effects.

The active, bilious purging, in some of these cases, caused by a single dose of half a grain of calomel, in intimate combination with a grain of sugar, is rather startling to one who for the first time observes it, and calls for an explanation which the writer is not now prepared to offer. The naked fact, however, is already established, and may be verified anywhere.

The dose of the compound, when used as a purgative, is the same for all ages and conditions, and, as will be observed, is more certain and uniform with adults than with children.

A considerable number of cases of habitual constipation have been treated with this powder, in minute doses, representing from one-eighth to one-sixteenth of a grain of the calomel, repeated every night, and continued for some weeks. By this means the evacuations have become healthy and regular, and at times excessive, requiring the suspension, occasionally, of even these minute doses.

In triturating the calomel and sugar together, it is observed that a *delicate canary color* is developed, which is partly lost again by keeping, and it is also observed that the activity of the powder is much increased by long trituration, thorough comminution, and intimate blending of the ingredients.

To the inquiry, "Whence this unwonted activity imparted to calomel by its trituration with cane sugar?" it may be answered, that the yellow color developed in the trituration raises a presumption of chemical reaction between the calomel and sugar; but upon the other hand, chemical analysis shows that there is no conversion of calomel into corrosive sublimate, or other mercurial salt soluble in water. Is not the yellow color due to an electrical penomenon consequent upon the trituration of the sugar? The fact that the color fades gradually by standing, favors this view,

A slight yellow color is produced in the trituration of calomel with milk sugar, but it quickly disappears.

It is proper that I should disclaim the discovery of this combination, for which I am indebted to my friend Dr. Miller, of Atlanta, Ga. Whether it is original with him or not, I am unable to say.

---

To Purify Drinking Water in cholera neighborhoods, the most simple and effectual way is to boil it. The air which has been expelled by the process may be added again by simply agitating the water in a bottle or pitcher, or by passing it two or three times through a fine sieve.

